# Variations of gastric corpus microbiota are associated with early esophageal squamous cell carcinoma and squamous dysplasia

**DOI:** 10.1038/srep08820

**Published:** 2015-03-06

**Authors:** Dariush Nasrollahzadeh, Reza Malekzadeh, Alexander Ploner, Ramin Shakeri, Masoud Sotoudeh, Saman Fahimi, Siavosh Nasseri-Moghaddam, Farin Kamangar, Christian C. Abnet, Björn Winckler, Farhad Islami, Paolo Boffetta, Paul Brennan, Sanford M. Dawsey, Weimin Ye

**Affiliations:** 1 Department of Medical Epidemiology and Biostatistics, Karolinska Institutet, Stockholm 17177, Sweden; 2 Digestive Oncology Research Center, Digestive Diseases Research Institute, Tehran University of Medical Sciences, Tehran, Iran; 3 Department of Public Health Analysis, School of Community Health and Policy, Morgan State University, Baltimore, Maryland, USA; 4 Division of Cancer Epidemiology and Genetics, National Cancer Institute, Bethesda MD 20892-7335, USA; 5 Institute for Translational Epidemiology and Tisch Cancer Institute, Icahn School of Medicine at Mount Sinai, NY 10029-6574, USA; 6 International Agency for Research on Cancer, Lyon, France

## Abstract

Observational studies revealed a relationship between changes in gastric mucosa and risk of esophageal squamous cell carcinoma (ESCC) which suggested a possible role for gastric microbiota in ESCC carcinogenesis. In this study we aimed to compare pattern of gastric corpus microbiota in ESCC with normal esophagus. Cases were included subjects with early ESCC (stage I–II) and esophageal squamous dysplasia (ESD) as the cancer precursor. Control groups included age and sex-matched subjects with mid-esophagus esophagitis (diseased-control), and histologically normal esophagus (healthy-control). DNA was extracted from snap-frozen gastric corpus tissues and 16S rRNA was sequenced on GS-FLX Titanium. After noise removal, an average of 3004 reads per sample was obtained from 93 subjects. We applied principal coordinate analysis to ordinate distances from beta diversity data. Pattern of gastric microbiota using Unifrac (p = 0.004) and weighted Unifrac distances (p = 0.018) statistically varied between cases and healthy controls. Sequences were aligned to SILVA database and *Clostridiales* and *Erysipelotrichales* orders were more abundant among cases after controling for multiple testing (p = 0.011). No such difference was observed between mid-esophagitis and healthy controls. This study is the first to show that composition of gastric corpus mucosal microbiota differs in early ESCC and ESD from healthy esophagus.

Cancer of the esophagus affects more than 450,000 people each year, of which 90% are squamous cell carcinomas (ESCC)^1^. Highest incidence rates have been reported from the “esophageal cancer belt”, an area that stretches from northern China to northern Iran^2^. The relationship between gastric environment and ESCC has been evaluated through observational studies^3^,^4^. As a link for this impact, atrophic gastritis has been shown to be associated with ESCC risk^5^, although no dose-response relation with severity of atrophy has been reported^6^. Human stomach was considered an inhospitable environment for bacteria until the recognition of *H. pylori*^7^ and most of research was focused on the relation between *H. pylori* and ESCC^5^,^8^,^9^,^10^,^11^,^12^,^13^,^14^ with showing little evidence of risk. Gastric acidity was believed to be a barrier against colonizing most of bacteria in the stomach, although 16S rRNA sequencing of gastric mucosa reveals a diverse bacterial community^15^. More than one hundred phylotypes have been detected in stomach of which 50% were from uncultivated bacteria^15^. Furthermore sequencing-based methods showed lower bacterial diversity associated with higher gastric pH^16^ even in the absence of atrophic gastritis. Even in *H. pylori* negative stomach high abundance of *Streptococcus* and *Prevotella*^17^ were observed, among them certain *Streptococcus* species are resistant to low pH^18^. Although risk of ESCC in relation with individual microorganisms has been tested, to date the association between gastric microbiota and ESCC has not been investigated.

Golestan province in Northern Iran is located in “esophageal cancer belt”^19^,^20^. We aimed to investigate associations between gastric mucosal microbiota and ESCC in this population. In this study in addition to early-stage ESCC cases and controls with healthy esophagus, we included mid-esophagitis as a diseased control group with inflammatory lesion in the esophagus and squamous dysplasia as the only known precursor of ESCC to compare the pattern of gastric microbiota.

## Results

Table 1 summarizes characteristics of the subjects and the reads. There was no significant differences between cases and controls except for the illiteracy rate which diseased-controls were less illiterate than cases and healthy controls (p = 0.001). Tobacco and alcohol consumption are not major risk factors in study area and there was no difference in proportion of exposed cases and healthy controls in our study samples. Table 2 summarizes major histopathologic findings in gastric biopsy samples. There was no difference in proportion of nonatrophic gastritis and intestinal metaplasia between cases and healthy controls. No evidence of gastric corpus atrophy was observed among study subjects.

**Figure 1:**
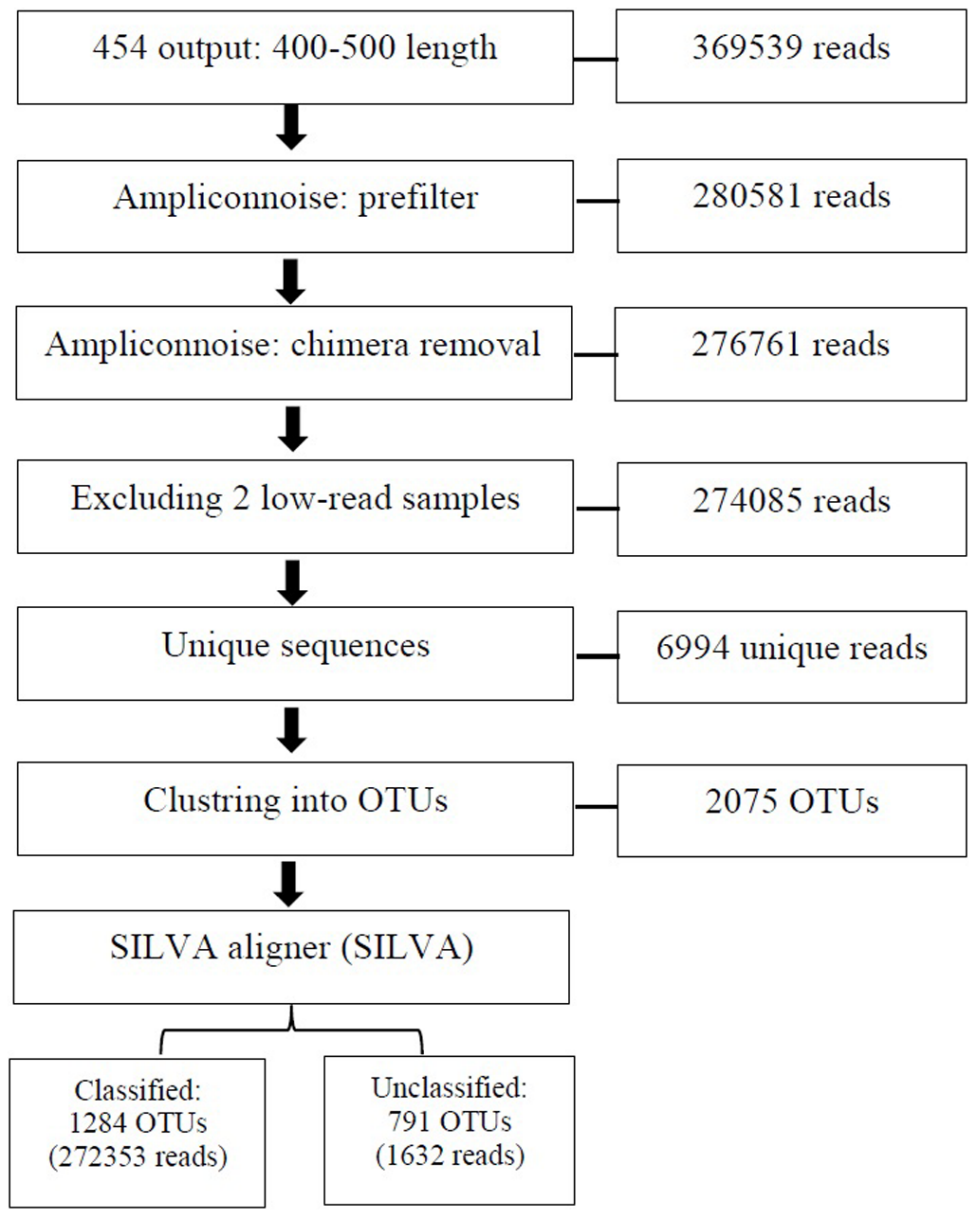
Sequence data processing from filtered reads to operational taxonomic units (OTUs) formation.

Figure 1 depicts sequence data processing steps. Briefly a total of 369,539 sequences with mean length of 419 nt from 93 tissue samples were evaluated (3004 average sequences per sample). A total of 25% of sequences was removed as noise or chimera. Two samples had less than 1000 reads (504 and 710 reads) and were excluded. Clusters of unique sequences at 3% of dissimilarity rate formed 2075 operational taxonomic units (OTUs). Of these OTUs, 1283 were assigned to bacterial taxa based on the SILVA database. The majority of unclassified OTUs (80%) appeared only once (n = 517) or twice (n = 123). The mean percentage of unknown OTUs per sample was 0.5%.

OTUs were assigned to 31 Phyla, 53 Classes, 90 Orders, 168 Families, and 336 Genera. Five most abundant phyla were *Firmicutes*, *Bacteroidetes Proteobacteria*, *Actinobacteria*, and *Fusobacteria*. Phyla composition was consistent across ESCC, ESD, healthy esophagus and mid-esophagitis groups. Table 3 summarizes the percentage of OTUs assigned to Order level among study cases and controls. Testing for differences in OTU abundance revealed higher abundance of *Clostridiales* (FDR = 0.011) and *Erysipelotrichales* Orders (FDR = 0.011) in the case group compared to the healthy esophagus group (Table 3). *Helicobacteriacea* composed nearly 43% of total reads amongst reads and all subjects except 3 cases and 2 controls had non-zero reads. Grouping abundance of *Helicobacteriacea*, no distinct cluster among cases was formed in relation to the quartile of reads. Alpha diversity measured by Chao1 was not significantly different among study groups though *Helicobacteraceae* abundance among cases (mean = 1059) was lower than healthy controls (mean = 1449) (p = 0.03) and diseased-controls (mean = 1715) (p = 0.02).

**Figure 2:**
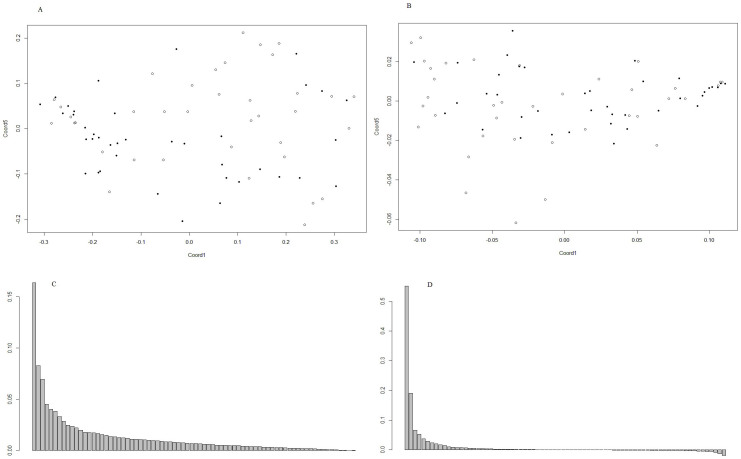
Distribution of samples depicted by PCoA coordinates for Unifrac (A) and weighted Unifrac distances (B). (Filled circles: cases, empty rings: healthy controls) Variations in distance matrix explained by coordinates based on Unifrac (C) and weighted Unifrac (D).

Unifrac and weighted Unifrac distances were ordinated by applying PCoA. Figure 2, depicted the percentage of variance coverage by Unifrac coordinates. Conditional logistic regression model was used to compare coordinates between cases and controls (Table 4). For Unifrac distance, a best model with three coordinates showed significant difference between cases and healthy esophagus group (*p* = 0.003) based on 37 matched pairs. Similar difference was found when OTU abundances were counted in the weighted Unifrac distances (*p* = 0.018). Removing ESCC cases and restricting the analysis to esophageal squamous dysplasia, did not change the results (based on 17 matched pairs). There was no individual taxonomic difference between the diseased-controls and healthy controls. We did not observe a significant difference in the coordinates based on Unifrac distances between diseased-control and healthy-control groups.

## Discussion

We have observed significant differences in microbiota composition of gastric fundal mucosa in subjects with early ESCC and ESD compared to those with a normal esophagus.

So far no single or combination of environmental or genetic risk factors has been identified to explain the high incidence of ESCC in Asian Cancer belt. Although alcohol and tobacco consumption account for major proportion of the disease in low risk area, their contribution to the risk of ESCC in high incidence regions of Asian Cancer Belt – from where 90% of cases arise- is limited. One suggested possible explanation for this excess risk is intrinsic exposure to carcinogens. Gastric mucosal changes have been shown to be associated with ESCC risk and bacterial alteration in the stomach has been considered to be the possible link. This study is the first to evaluate the relevance of microbial link in this association.

Similar to studies on gastric mucosa microbiota^15^, the most common phyla in our samples were *Proteobacteria*, *Firmicutes*, *Bacteroidetes*, *Actinobacteria*, and *Fusobacteria*. Our data suggests that presence and abundance of *Clostridiales* and *Erysipelotrichales* Orders were associated with early ESCC and ESD. Both microbial hints belong to *Firmicutes* phylum. In an animal study, presence of members of *Clostridiales* altered the pathogenicity of *H. pylori* by recruitment of CD_4_ T-cells to the gastric mucosa^21^ which suggests a possible impact of microbial composition on gastritis outcome. Higher abundance of *Clostridiales* might enhance more aggressive response to *H. pylori* and induces severe or pangastritis which may further develop to atrophic gastritis. The link between *Clostridiales* and the esophagus has been observed in response to proton pump inhibitor use, as abundance of its members increases in the lower esophagus. Such an effect has not been reported in gastric mucosa. *Erysipelotrichales* is highly dependent on the fat content in the diet of humanized mice model^22^, associated with periodontitis^23^ and gut inflammatory response in animal model^24^. No study has been published on its association with esophageal disorders, but its high abundance in periodontitis microbiota and the observed association of poor oral heath with ESCC risk^25^ may be one explanation for its role and our finding. Difference between diversity of oral microbiota in ESD patients compared to those with healthy esophagus has been shown in a cross-sectional study^26^.

The relation between *H. pylori* and ESCC risk is controversial. Most studies have not shown an excess risk including in our study population^27^ with more than 80% *H. pylori* seropositivity^27^. We observed lower abundance of *Helicobacteriacea* order among case group which might be secondary to subclinical atrophic changes in the stomach. Our finding on non-distinct microbial clusters in gastric mucosa in relation to different abundances of *H. pylori* among ESCC is similar to the report in healthy stomach^15^. Although given that the *H. pylori* infection is prevalent in catchment area (seropositivity >84% among older >55 years), any inference on relation between *H. pylori* and gastric microbiota in this study should be done with caution.

In this study, 16S rRNA with coverage of 464 nt of region V3–V4 was used to distinguish bacteria. Although it did not cover whole hyper variable V1–V9 regions, the most divergent regions were covered and it was possible to align with SILVA reference with 90.5% coverage. Furthermore, V4 and V5 are less specific for *species* level^28^ and we avoided assigning *genus* or *species* nomenclature to the OTUs. Although 16S is a gold standard for bacterial phylogeny, presence of its multiple copies in some bacteria with slight difference in sequence could lead to identifying multiple types of the same bacterium^29^. As our finding of different microbial composition did not rely only on presence or absence of taxa, possible discriminatory power of 16S for bacterial detection would not make limitation on inferring difference in this study.

Some of gastric microbiota are anaerobes and difficult to cultivate and some of them could not be assigned to reference databases. We tried to reduce this effect through OTU formation. In our data unassigned OTUs were unevenly distributed among samples, as five samples shared most of them. These rare species could be formed due to intrinsic errors of pyrosequencing^30^ though per-base error rate is comparable or lower for 454 sequencing compared to sanger sequencing^31^.

Modest sample size is one of the limitations of this study, though based on national cancer registry data less than 9% of ESCC cases are diagnosed at early stages^32^. In addition squamous dysplasia is a rare phenomenon even in high risk areas for ESCC, as its prevalence among endoscopy subjects is less than 5%^33^. Due to this we combined early ESCC and ESD as one case group. Though is probable that the low number of study subjects was in part compensated for by the higher depth of sequencing. Previous studies have considered a broad range of reads (from 100 to 10,000) to be sufficient depth to separate the microbiota of healthy and diseased organs^34^, though higher sequencing depth might amplify the difference in our study. However we did not observe difference in alpha diversity between healthy-controls and cases, we do not think that the depth of sequencing was enough for estimating alpha diversity of microbiota. It has been shown that at least 5000 clean reads are needed for valid estimation of diversity^35^.

Similar to every PCR-based methods, amplification bias is a concern as some less abundant taxa might be underestimated or ignored. Using endoscopy clinic controls may limit the generalizability of our results to healthy populations, although several biopsies from the esophagus were carefully examined before assigning subjects to healthy control group.

We used mid-esophagus esophagitis as a diseased-control to evaluate the specificity of our findings. Inflammation is a common pathway between neoplasia and esophagitis meanwhile esophagitis does not play a role in ESCC carcinogenesis. Detection of explicitly different microbiota pattern between cases and healthy controls and not with diseased-controls increases the validity of our findings. Number of esophagitis controls was less than healthy controls and 16 out of 17 were male, although no difference in microbiota was observed between male and female, reduced sample size might affect the precision of statistical tests.

In this study – similar to the most other studies- we used one snap frozen biopsy from mid corpus of the gastric greater curvature. In an early study of gastric biota^15^, it has been shown that phylogenic pattern of mucosal biota did not differ between the corpus and antrum, although corpus mucosal microbiota may not be representative of whole gastric microbiota.

The different patterns of gastric microbiota that we observed might be secondary to cancer. We used early-stage ESCC and patients with ESD (the asymptomatic precursor lesion of ESCC) as cases, to decrease this possibility. As a sensitivity test we excluded Early-stage ESCC and observed different microbiota pattern between healthy controls and ESD subjects (data was not shown), which lowered the possibility that our findings is secondary to cancer. Although it is probable that microbiota pattern changes secondary to dysplasia.

In summary we observed an altered gastric corpus microbiota in patients with early ESCC or ESD, compared to subjects with healthy esophagus, with higher abundance of bacteria in the *Clostridiales* and *Erysipelotrichales* Orders. Studies with higher sequencing depth and larger sample sizes are warranted.

## Methods

### Ethical approval

From all subjects, an informed consent was obtained. The case-control study was approved by the local ethical committee in the Digestive Disease Research Centre of Tehran University of Medical Sciences, Iran (IRB00001641), Institutional Review Board of National Cancer Institute, US and Stockholm Regional Ethics Vetting Board, Sweden (Dnr:2010/471-31/4). The methods were carried out in accordance with the approved guidelines.

### Subjects

Details of study design have been reported earlier^36^. Briefly, subjects were recruited at Atrak clinic, the only specialized clinic for upper gastrointestinal cancer diagnosis and treatment in eastern Golestan, from December 2003 to June 2007. During study period, all physicians in catchment area were contacted and asked to refer patients with clinical suspicion for upper gastrointestinal malignancies to this clinic. For all subjects, upper gastrointestinal endoscopy was performed and at least 9 biopsies from the normal-looking esophagus and stomach were taken. To find squamous dysplasia, esophageal chromoendoscopy with 2% Lugol solution was done and biopsies were taken from unstained mucosa. Unstained lesions were further examined in Digestive Disease Research Center, Tehran University. Those with squamous dysplasia and no evidence of cancer were included as esophageal squamous dysplasia (ESD) subjects. Based on cancer registry data, during the study period it was estimated that about 70% of incident ESCC cases in catchment area were recruited. The majority of ESCC patients (>90%) were diagnosed with clinical Stage III–IV disease at the time of recruitment. To reduce the chance that differences in the gastric microbiota might be secondary to cancer, we included patients who were diagnosed with clinical Stage I–II ESCC plus all patients who were diagnosed with ESD during study in our case group. Two control groups were randomly selected from endoscopy clinic patients who had the same referral pattern as cases, including (1) healthy controls with endoscopically and histologically normal esophagus in all biopsies and (2) diseased controls with histologic esophagitis in mid-esophageal biopsies.

Sample size of original case-control study was calculated based on achieving power of 95% to detect odds ratio of 2 between matched cases and control and total of 300 cases and 600 controls were calculated. For present study, the difference between 2 taxonomic levels of more than 20% or odds ratio of 2.5 would require at least 30 pair of case-control to achieve 90% power. A priori for odds ratio was based on reports from other studies in gastrointestinal malignancies and microbiota^37^.

Among 579 endoscopy clinic controls and 300 ESCC cases, we found 19 early-stage ESCC and 18 ESD patients. One age/sex-matched control was randomly selected from endoscopy clinic controls with a healthy esophagus for each case. We also found 17 subjects with mid-esophageal esophagitis who were age and sex matched with the cases, and included them as a diseased control group. For each subject, one snap-frozen biopsy from mid corpus of the stomach greater curvature was preserved in RNA-later preserved at −70 and send to Karolinska Institutet on dry ice for microbiota assessment.

### DNA extraction and sequencing

DNA was extracted from snap frozen gastric tissue (approximately 5–6 mm in length) using DNeasy Blood & Tissue Kit (Qiagen. Inc., Valencia. CA). Tissues were treated with filtered-lysozyme in lysis buffer (Tris-EDTA-Triton) and overnight incubation at 56°C in buffer AL (Qiagen. Inc., Germany) and proteinase K and blended with glass beads (Tactum Lab. Sweden) with 0.1. 0.5 and 1 mm diameter for 1 minute in Bullet Blender (BBX24. Next Advance. Inc., NY). Mixture was incubated with RNase (Qiagen. Inc., Valencia. CA). Tubes containing only beads, lysozyme, lysis buffer, and extraction kit substances were included as quality controls for contamination.

Small subunit of ribosomal RNA (16 S rRNA) gene was amplified from extracted DNA by using primers targeting region V3–V4. Forward primer (Bekt_341F: 5′-CCTACGGGNGGCWGCAG) and reverse primer (Bekt_805R: 5′-GACTACHVGGGTATCTAATCC) carried 454-adaptor sequences A and B^38^. For multiplexing, unique 7 bp barcode for each sample was included in reverse primer in a way that barcodes differ in 2 nucleotides. Polymerase chain reaction (PCR) included a mix containing 10 μl 5X PCR buffer HF. 0.65 μl Phusion high fidelity DNA polymerase (New England Biolab Inc.. MA), 1 μl PurePeak deoxyribonucleoside triphosphates (200 μM. ThermoScientific. Milwaukee), and 2.5 μl of each primer (MWG eurofins. Germany). Two μl of template DNA was added to this mix with total volume of 50 μl, GeneAmp PCR system 9700 cycler (Applied Biosystems. CA) was used with cycling parameters of initiation (95°C for 5 min), 30 cycles of denaturation (95°C for 40 s), annealing (58°C for 40 s), and elongation (72°C for 1 min) with a final extension at 72°C for 7 min. Each sample was put in triplet with one negative control for avoiding PCR cross contamination. Amplified products for each sample were pooled and verified by gel electrophoresis. PCR products were purified by Agencourt AMPure XP magnetic beads (Beckman Coulter, Inc.) with size selection through modifying PEG concentration to obtain 400–500 nt template. DNA concentration in each sample was measured using Qubit 2.0 fluorometer (Invitrogen Inc., UK). An amplicon pool was formed by pooling equimolar amounts of all DNA libraries for a minimum of 20 ng/dl. Pool amplicon was subsequently sent to SciLifeLab (Stockholm), where pooled DNA was amplified in PCR-mixture-in-oil emulsions and sequenced on whole PicoTiterPlate (PTP) on GS-FLX Titanium XLR-70 system (Roche Diagnostics Co. Sweden). Plate was physically divided into 2 lanes and duplicate samples were run on both lanes.

### Sequence data processing

Standard flowgrams were split to individual samples. AmpliconNoise v1.25 with its default filtering setting (PCR noise precision = 30. pyro noise precision = 0.6) was used to remove noisy data^39^. Chimeric sequences were removed by applying Perseus^39^. All noise-free individual sequences were combined and clustered by applying complete linkage clustering algorithm in FCluster. Clusters were remapped to operational taxonomic units (OTUs) at the similarity level of 97%. The taxonomic position of the representative sequence of each OTU was identified using last common ancestor method implemented in SINA aligner^40^ which was run against SILVA SSU 111 reference database^41^ imported to ARB^42^.

### Statistical analysis

Two sets of analysis were done:(a) Individual taxonomy comparison: Assigned OTUs were compared individually between study groups using conditional logistic regression with false discovery rate (FDR) control to correct for multiple comparisons^43^. To have power for analysis, a minimum of 8 reads per OTU were required. (b) Microbiota pattern analysis, including all assigned and unassigned OTUs: Unifrac and weighted Unifrac distances^44^ were fed into a principal coordinate analysis (PCoA). Conditional logistic regression and Akaike information criterion (AIC) were applied to select the best-fit model. To examine whether presence or abundance of *H. pylori* influences the microbiota composition, an average-linkage hierarchical clustering analysis was performed excluding *H. pylori* from microbiota and proportion of different quartiles of *H. pylori* was compared between clusters. R and phyloseq package were used for statistical analysis (www.r-project.org)^43^.

## Author Contributions

Conception and design: W.Y., D.N., R.M. Development and Methodology: D.N. and W.Y. Acquisition of data: D.N., M.S., R.M., S.N.M., R.S., S.F. and F.I. Laboratory analysis: D.N. and W.Y. Analysis and interpretation of data: D.N., A.P. and W.Y. Drafting Manuscript: D.N. Review, revision of manuscript: W.Y., D.N., B.W., A.P., R.M., S.D., C.A., F.K., P.Bo. and P.Br. Study Supervision: W.Y. and R.M.

## Figures and Tables

**Table 1:** Characteristics of the subjects and microbiome reads among study groups

	Cases (ESCC and ESD)	Healthy Controls (normal esophagus)	Diseased controls (mid-esophageal esophagitis)
**Subjects**			
Number of subjects	37	37	17
Male/female	18/19	18/19	16/1
Mean age (SD)	64.5 (11.8)	62.1 (16.3)	63.6 (14.0)
Rural residence (%)	25 (69)	19 (54)	6 (35)
Illiterate (%)	30 (81)	28 (76)	6 (35)
Ever tobacco use (%)	14 (38)	12 (32)	10 (59)
Ever alcohol use (%)	1 (2.7)	1 (2.7)	1 (5.9)
Ever opium use (%)	11 (29.7)	14 (37.8)	8 (47)
Mean DMFT score (SD)	27.8 (6)	25 (9)	25 (10)
**Reads diversity**			
Mean sequences	2969	2875	3047
Mean Chao1	191	178	154
Mean Shannon's	2.4	1.4	1.9

ESCC: esophageal squamous cell carcinoma; ESD: esophageal squamous dysplasia; DMFT: diseased, missing and filled teeth.

**Table 2:** Histopathologic characteristics of the gastric corpus among study groups

Gastric corpus histology	Cases (ESCC and ESD)	Healthy controls (normal esophagus)	Diseased controls (mid-esophageal esophagitis)
**Gastritis**			
Normal	21 (56.7)	19 (51.3)	5 (29.4)
Chronic/active gastritis			
Moderate	15 (40.6)	17 (46.0)	11 (64.6)
Severe	1 (2.7)	1 (2.7)	1 (6.0)
Atrophic gastritis			
Moderate/Severe	0	0	0
**Metaplasia**			
None	33 (89.2)	32 (86.4)	12 (70.6)
Intestinal	4 (10.8)	5 (13.6)	5 (29.4)

**Table 3:** Percentage of OTUs assigned to the Order level of taxonomy. OTUs with abundance of eight and less were not included.(cases: early esophageal squamous cell carcinoma and esophageal dysplasia, diseased-control: mid-esophageal esophagitis)

Order	Cases (N = 37)	Healthy controls (N = 37)	P-value	FDR*	Diseased control (N = 17)	P-value	FDR*
Actinomycetales	2.63	2.20	0.351	0.556	2.61	0.468	0.799
Bacillales	1.50	2.75	0.527	0.645	1.31	0.995	0.995
Bacteroidales	18.80	21.98	0.013	0.147	24.84	0.257	0.738
Bifidobacteriales	2.26	1.65	0.019	0.158	1.31	0.830	0.951
Burkholderiales	1.50	1.65	0.210	0.525	1.31	0.862	0.951
Campylobacterales	1.50	2.20	0.057	0.311	2.61	0.524	0.800
CandidatedivisionOD1/SR1/TM7	3.01	2.75	0.575	0.655	2.61	0.866	0.951
Caulobacterales	0.38	0.55	0.500	0.634	-	-	-
Clostridiales	27.82	14.84	0.001	0.011	16.99	0.167	0.738
Coriobacteriales	1.13	1.10	0.359	0.556	1.31	0.034	0.716
Corynebacteriales	1.13	0.55	0.183	0.525	0.65	-	-
Enterobacteriales	1.88	0.55	0.371	0.556	0.65	-	-
Erysipelotrichales	1.50	0.55	0.001	0.011	0.65	0.298	0.738
Firmicutes order	0.38	0.55	0.054	0.311	0.65	0.356	0.738
Flavobacteriales	3.38	3.85	0.088	0.362	1.96	0.523	0.800
Fusobacteriales	5.26	7.69	0.266	0.556	5.23	0.116	0.738
Lactobacillales	8.65	13.19	0.695	0.764	14.38	0.912	0.951
Micrococcales	1.13	1.65	0.889	0.889	1.96	0.900	0.951
Mycoplasmatales	0.38	1.10	0.315	0.556	0.65	-	-
Neisseriales	1.50	2.20	0.498	0.634	2.61	0.230	0.738
Pasteurellales	4.14	6.04	0.216	0.525	6.54	0.417	0.755
Pseudomonadales	3.38	3.30	0.184	0.525	3.27	0.318	0.738
Rhizobiales	0.75	0.55	0.576	0.655	-	-	-
Sphingobacteriales	0.75	1.10	0.387	0.556	1.31	0.918	0.951
Unclassified order	2.26	3.30	0.727	0.774	1.31	0.679	0.951
Xanthomonadales	1.13	1.65	0.223	0.525	1.96	0.074	0.716

OTU: operational taxonomic unit; FDR: False discovery rate. In total, 1.90% of microbiota among cases, 0.55% among healthy controls, and 1.31% among diseased controls belonged to unique orders.

**Table 4:** Conditional logistic regression models of coordinates based on Unifrac and weighted Unifrac distances. Models compared cases (Early ESCC and ESD) with matched healthy controls

	Unifrac distance				Weighted Unifrac distance			
	Coordinate No.	AIC	*P*-value for likelihood ratio* test	*P*-value for testing coordinates	Coordinate No.	AIC	*P*-value for likelihood ratio test	*P*-value for testing coordinates
Full model	10	47.85	0.0247	---	10	55.09	0.139	---
Best model	3	42.35	0.00358	---	3	45.84	0.018	---
Best model components	Coordinate 1	---	---	0.013	Coordinate 1	---	---	0.019
	Coordinate 4	---	---	0.150	Coordinate 3	---	---	0.160
	Coordinate 5	---	---	0.038	Coordinate 5	---	---	0.100

AIC: Akaike information criterion.

*P-values of comparing null model (without coordinates) to full model and best model.
